# Role of Interleukin-1 Family Members and Signaling Pathways in KSHV Pathogenesis

**DOI:** 10.3389/fcimb.2020.587929

**Published:** 2020-10-30

**Authors:** Lindsey Barrett, Jungang Chen, Lu Dai, Karlie Plaisance-Bonstaff, Luis Del Valle, Zhiqiang Qin

**Affiliations:** ^1^ Department of Pathology, Winthrop P. Rockefeller Cancer Institute, University of Arkansas for Medical Sciences, Little Rock, AR, United States; ^2^ Department of Medicine, Louisiana State University Health Sciences Center, Louisiana Cancer Research Center, New Orleans, LA, United States; ^3^ Department of Pathology, Louisiana State University Health Sciences Center, Louisiana Cancer Research Center, New Orleans, LA, United States

**Keywords:** Kaposi’s sarcoma-associated herpesvirus, Kaposi’s sarcoma, primary effusion lymphoma, multicentric Castleman’s disease, interleukin-1

## Abstract

Kaposi’s sarcoma-associated herpesvirus (KSHV) represents the etiological agent for several human malignancies, including Kaposi’s sarcoma (KS), primary effusion lymphoma (PEL), and multicentric Castleman’s disease (MCD), which are mostly seen in immunocompromised patients. In fact, KSHV has developed many strategies to hijack host immune response, including the regulation of inflammatory cytokine production. Interleukin-1 (IL-1) family represents a major mediator for inflammation and plays an important role in both innate and adaptive immunity. Furthermore, a broadening list of diseases has revealed the pathologic role of IL-1 mediated inflammation. In the current mini-review, we have summarized recent findings about how this oncogenic virus is able to manipulate the activities of IL-1 signaling pathway to facilitate disease progression. We also discuss the therapeutic potential of IL-1 blockade against KSHV-related diseases and several unsolved questions in this interesting field.

## Introduction

Kaposi’s sarcoma-associated herpesvirus (KSHV) infection causes several human cancers including Kaposi’s sarcoma (KS), primary effusion lymphoma (PEL), and multicentric Castleman’s disease (MCD) (Broussard and Damania, 2019). These KSHV-associated malignancies develop mainly in immunocompromised patients, especially those infected with human immunodeficiency viruses (HIVs) (Vangipuram and Tyring, 2019). Further, the morbidity rate of patients with KSHV-associated diseases is much higher in patients with compromised immune systems compared to those with competent immune systems (Mesri et al., 2010).

KSHV has two alternating life-cycle programs following primary infection of host cells, the latent and lytic phases, which are characterized by different patterns of viral gene expression (Mesri et al., 2010). During latency, viral genomes persist as circular episomes with no progeny virion production and only a limited number of latency-associated genes expressed, including latency-associated nuclear antigen (LANA), viral Fas-associated protein with death domain (FADD)–like interleukin-1β-converting enzyme (FLICE)-like inhibitory protein (vFLIP), viral cyclin (vCyclin), as well as some viral microRNAs (Uppal et al., 2014). Once entering the lytic phase, which is caused by various stimuli, almost all viral genes are highly expressed, followed by genomic DNA replication and mature virion release (Ye et al., 2011). KSHV is known to hijack many aspects of the host’s immune response such as viral detection and cytokine production. Interleukin-1 (IL-1) is an inflammatory cytokine family of 11 distinct proteins that has a wide array of functions in innate immunity processes. The IL-1 superfamily contains many pro-inflammatory cytokines (IL-1α, IL-1β, IL-18, IL-33, IL-36α, IL-36β, and IL-36γ) and a few anti-inflammatory cytokines (IL-36Ra, IL-37, and IL-38) (Boraschi and Tagliabue, 2013). Among them, IL-1 is the defining member of this family and its physiology and relationship to pathology has been thoroughly studied and reported. IL-1 includes two activator cytokines, IL-1α and IL-1β, and one inhibitory factor, the IL-1 receptor antagonist (named as IL-1Ra). The main function of IL-1 is to respond to tissue damage caused by pathogen-associated molecular patterns (PAMPs) such as viral products, or damage-associated molecular patterns (DAMPs) such as adenosine 5’-triphosphate (Xu et al., 2019). Upon stimulation, IL-1α and IL-1β both bind to the type I IL-1 receptor (IL-1R1) which then recruits the IL-1 receptor accessory protein (IL-1RAP), as well as the adaptor protein MyD88, which are necessary for triggering signal transduction. Once the IL-1 receptor complex is formed, a downstream signaling cascade is activated which then stimulates a collection of related immune responses and/or inflammatory genes (Jensen, 2017). Dysregulation of the IL-1 pathway has been shown to be linked to a number of autoinflammatory and autoimmune diseases, such as atherosclerosis and systemic sclerosis, respectively, as well as cancers like gastric carcinoma and lung cancer (El-Omar et al., 2001; Bhat et al., 2014; Ridker et al., 2017; Xu et al., 2019).

KSHV infection has been found to induce the production of a variety of host pro-inflammatory cytokines. For example, primary KSHV infection in monocytes can increase the release of IL-1α, IL-1, and IL-6 (Host et al., 2017). These cytokines have been suggested to regulate early KS lesion progression and have been found at high levels in the sera of KS patients (Ensoli and Stürzl, 1998). Other IL-1 family members such as IL-33 have recently been shown to play a role in KSHV pathogenesis by regulating chromatin compaction through nucleosome-nucleosome interactions (Roussel et al., 2008). Therefore, in this mini-review, we will summarize recent findings about the relationship between KSHV and the IL-1 family members. We will try to highlight how KSHV may utilize the IL-1 signaling pathway to facilitate disease progression and how potential immunotherapies could target such mechanisms.

## The IL-1 Signaling Pathway

IL-1 is a major mediator for inflammation and plays an important role in both innate and adaptive immunity. IL-1α and IL-1β both signal through the cell surface receptor, IL-1R1 (Jensen, 2017). Upon ligand binding, the transmembrane IL-1R accessory protein, IL-1RAP, is recruited to the site. This heterodimer formation leads to intracellular recruitment of the adaptor protein, MyD88, and mobilization of IL-1R-associated kinases (e.g., IRAK1, IRAK2, and IRAK4). These kinases, along with additional signaling factors, lead to the phosphorylation and degradation of nuclear factor κB (NF-κB) inhibitor IκB. The end of this signaling pathway results in the translocation of activated transcription factors, such as activator protein 1 (AP-1) and NF-κB, to the nucleus where specific gene expression is activated. IL-18 and IL-33 also stimulate gene expression through the same intracellular pathway using their receptor-accessory protein complexes (IL-18R1/IL-18RAP and IL-1R-like 1/IL-1RAP). The other cytokines, IL-36α, IL-36β, and IL-36γ, bind to the receptor IL-1R-like 2 (IL-1RL2 or IL-36R), which then uses IL-1RAP as its receptor-accessory protein and stimulates the same signaling cascade as IL-1, IL-18, and IL-33 (Dinarello, 2019). Interestingly, KSHV has developed strategies to manipulate the functions of b1these different IL-1 signaling molecules after invading host cells.

## IL-1α/β and Receptors

**Figure 1:**
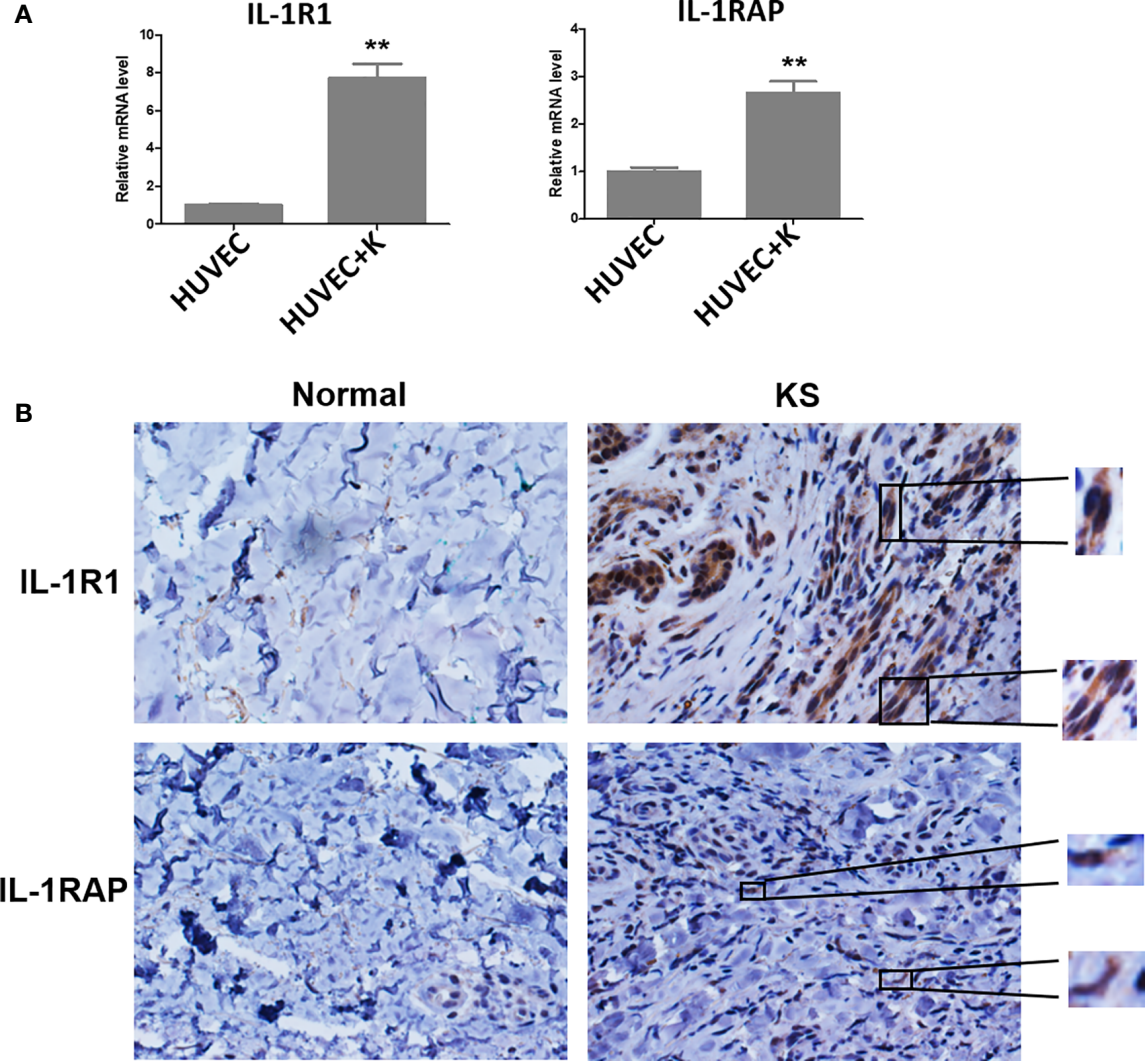
IL-1R1 and IL-1RAP are upregulated by KSHV and highly expressed within AIDS-KS tumor tissues. **(A)** Primary human umbilical vein endothelial cells (HUVEC) were infected with KSHV (MOI~10) or not for 48 h, followed by qRT-PCR analysis. The data were normalized to the β-actin housekeeping gene expression. Error bars represent the S.D. for three independent experiments. **p < 0.01. **(B)** Expression of IL-1R1 and IL-1RAP in representative formalin-fixed paraffin-embedded KS tissues from HIV+ patient without treatment were determined by immunohistochemical staining (40×). The higher magnification for IL-1R1 and IL-1RAP detection in KS tumor cells were also shown (60×). Normal skin tissues were used as a control.

Several studies have reported that KSHV infection or viral protein infiltration can upregulate IL-1α and/or IL-1β expression. For instance, one study showed that ectopic expression of viral macrophage inflammatory protein-II (vMIP-II) within endothelial cells upregulated multiple proangiogenic factors, including IL-1α, resulting in enhanced angiogenesis (Cherqui et al., 2007). Another viral protein, vOX2, a glycosylated cell surface protein, was found to dramatically stimulate primary monocytes, macrophages, and dendritic cells to produce IL-1β (Chung et al., 2002). On the other hand, KSHV Orf63, encoding a viral homolog of human NLRP1 (NACHT, LRR, FIIND, CARD domain and PYD domains-containing protein 1), was found to reduce IL-1β expression and related signaling through inhibition of the inflammasome (Gregory et al., 2011). In fact, many studies suggest that inflammatory and angiogenic cytokines including IL-1β contribute to the pathogenesis of KS by causing abnormal proliferation, angiogenesis, and a KS-like phenotype independent of KSHV (Ensoli et al., 1992). For example, IL-1β was markedly elevated in most KS lesions (Samaniego et al., 1997). Furthermore, IL-1β was elevated during initial KSHV-MCD flares compared with remission (Polizzotto et al., 2013). Interestingly, our recent study demonstrated that IL-1β was required for the upregulation of PD-L1 expression by viral lytic reactivation from KSHV-infected tumor cells (Chen et al., 2019), which may represent a novel mechanism for virus-associated tumor cell immune escape. It remains unclear about the situation of receptor and accessory proteins of IL-1 in KSHV-infected cells. Our recent data indicate that KSHV infection significantly upregulates IL1R1 and IL1RAP from endothelial cells (**Figure 1A**). Moreover, both proteins are found highly expressed in KS tumor cells, especially IL1R1 (**Figure 1B**).

## IL-18, IL-33, and IL-36

KSHV latency, especially viral FADD-like interleukin-1-β–converting enzyme [FLICE/caspase 8]-inhibitory protein (vFLIP), was found to induce the expression of IL-18 (as well as IL-1β) in an NF-κB dependent manner (Singh et al., 2013). In contrast, one of the viral lytic products, KSHV polyadenylated nuclear RNA (PAN RNA) decreased the expression of IL-18 (Rossetto and Pari, 2011). Similar inhibitory effects on IL-18 were observed with KSHV Orf63 in the study mentioned above (Gregory et al., 2011).

The functional roles of IL-33 and IL-36, and their regulatory mechanisms in KSHV-infected cells remain mostly unclear. One very recent study reported that the plasma IL-33 concentrations were higher in individuals with KS in Uganda, Africa (Byakwaga et al., 2020), implying that this cytokine and its related signaling may also play role in KSHV pathogenesis. Interestingly, IL-33 has also been demonstrated as a chromatin-associated factor in the nucleus of endothelial cells, which has a short chromatin-binding peptide that shares similarities with a motif found in KSHV-encoded latency-associated nuclear antigen (LANA) (Carriere et al., 2007; Roussel et al., 2008). As we know, LANA is responsible for the attachment of the viral episome to mitotic chromosomes (Barbera et al., 2006); thus, this IL-33 peptide can also dock into the acidic pocket formed by the H2A-H2B dimer at the nucleosomal surface and regulate chromatin compaction through nucleosome-nucleosome interactions. However, the association between IL-33 and KSHV latency and lytic reactivation remains unknown.

## MyD88 and IRAKs

One study using X chromosome-targeted sequencing identified 34 common missense mutations in 100% of PEL cases, including a Phe196Ser change in the IRAK1 protein. Moreover, IRAK1 was constitutively phosphorylated in PEL and required for tumor cell survival (Yang et al., 2014). By using CRISPR/Cas9 knockout technology, the same group recently reported that established PEL cell lines were able to circumvent the loss of IRAK1, IRAK4, and MyD88, while the deletion clones were deficient in IL-10 production (Seltzer et al., 2020). Due to the suppression of T cell function by IL-10, the authors suggest that the IRAK pathway may contribute to early-stage development of PEL. KSHV encodes 12 pre-microRNAs (pre-miRNAs), which are processed into 25 mature microRNAs (miRNAs) (Qin et al., 2017). Interestingly, Abend et al. reported that IRAK1 and MyD88 were directly targeted by several KSHV-microRNAs, particularly miR-K12-9 and miR-K12-5, respectively (Abend et al., 2012). The presence of miR-K12-9 and miR-K12-5 inhibited the production of IL-6 and IL-8 upon IL-1α stimulation of endothelial cells. In another study, Lingel et al. reported that KSHV-encoded replication and transcription activator (RTA) was able to bind to MyD88 RNA and suppress its RNA synthesis (Lingel et al., 2016). Another group recently found that KSHV RTA downregulated MyD88 expression at the protein level by degrading MyD88 through the ubiquitin (Ub)-proteasome pathway (Zhao et al., 2015).

## IL-1 Blockade

Since IL-1 is a master cytokine of local and systemic inflammation, pharmacological blockade of IL-1 activity has been applied in a variety of inflammatory diseases that results in a rapid and sustained reduction in disease severity. There are three major categories of IL-1 blockers which have been approved by the Food and Drug Administration (FDA) for clinical treatment: **1)** The IL-1 receptor antagonist (e.g., Anakinra), blocks the IL-1 receptor and therefore reduces the activity of IL-1α and IL-1β; **2)** The soluble decoy receptor (e.g., Rilonacept, also known as IL-1 Trap), a dimeric fusion protein consisting of the ligand-binding domains of the extracellular portions of IL-1R1 and IL-1RAP linked in-line to the fragment-crystallizable portion (Fc region) of human IgG1 that binds and neutralizes IL-1; **3)** The neutralizing monoclonal anti-IL-1β antibody (e.g., Canakinumab), which directly targets IL-1β (Dinarello and van der Meer, 2013; Dinarello, 2013). There are other neutralizing monoclonal antibodies targeting IL-1α or the IL-1 receptor in different clinical trials (Dinarello and van der Meer, 2013). Interestingly, Boehringer Ingelheim Company recently developed a new IL1RAP antibody, BI-5041, which targets a unique epitope on IL-1RAP and therefore blocks IL-1, IL-33, and IL-36 signalling. Currently, there is limited data about IL-1 blockade therapy in KSHV-related malignancies (El-Osta et al., 2010). Two case reports detailed the successful treatment of MCD by Anakinra for two patients, although their KSHV infection status remains unclear (Galeotti et al., 2008; El-Osta et al., 2010).

## Conclusion and Prospective

**Figure 2:**
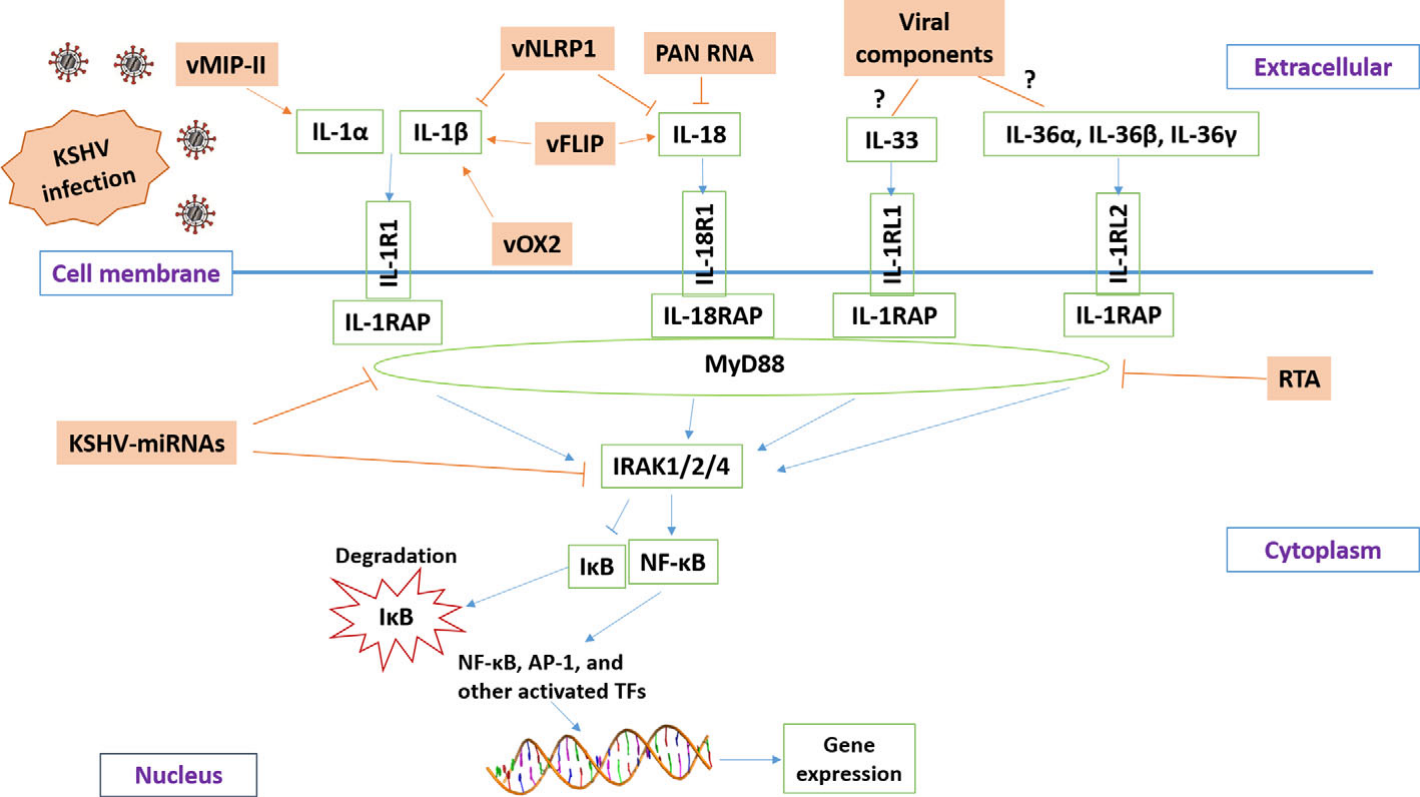
Schematic diagram of potential mechanisms for KSHV manipulating IL-1 signaling pathway. The brown and green rectangles represent viral and host genes, respectively. The arrows and bars represent the activation and inhibition, respectively. Notably, the mechanisms of KSHV regulation of IL-33 and IL-36 signaling remain mostly unknown.

Current research reveals that KSHV has developed different strategies to manipulate IL-1 signaling activity (summarized in **Figure 2**) in order to balance the host’s inflammatory response or help the virus escape the host’s immune response. The virus-encoded latent and lytic proteins and even viral non-coding RNAs can target multiple components of the IL-1 signaling pathway. However, there are still many questions in this field waiting for further investigation. For example, the functions and regulatory mechanisms of certain IL-1 family members (e.g., IL-36, IL-37, IL-1Ra, and IL-1RAP) during KSHV infection or virus-induced tumorigenesis remain unknown. It is also unclear whether the intermediates of IL-1 signaling may affect KSHV replication, especially the “latency to lytic” switch. Furthermore, the efficacy of IL-1 blockade therapy either alone or combined with other therapies for KSHV-related malignancies needs to be tested. Fully understanding these questions will shed light on the molecular mechanisms of KSHV pathogenesis and tumorigenesis and facilitate the development of more efficacious antiviral and anticancer treatments.

## Author Contributions

LB wrote the manuscript. JC, LD, and KP-B participated in figure preparation and discussion. LV and ZQ reviewed and edited the manuscript. All authors contributed to the article and approved the submitted version.

## Funding

This work was supported by the Arkansas Bioscience Institute, the major research component of the Arkansas Tobacco Settlement Proceeds Act of 2000, and Winthrop P. Rockefeller Cancer Institute Core Facility Voucher Award. Funding sources had no role in study design, data collection and analysis, decision to publish, or preparation of the manuscript.

## Conflict of Interest

The authors declare that the research was conducted in the absence of any commercial or financial relationships that could be construed as a potential conflict of interest.
